# Insular changes in autism spectrum disorder patients

**DOI:** 10.1192/j.eurpsy.2023.1083

**Published:** 2023-07-19

**Authors:** H. Arshad, A. Arshad, G. Muhammad, S. Khatri

**Affiliations:** 1 Psychiatry, Jinnah Sindh medical university, Karachi, Pakistan; 2 Psychiatry, Ocean Medical Center, New Jersey, United States

## Abstract

**Introduction:**

Autism Spectrum Disorder is a chronic medical condition usually diagnosed during childhood. With psychosocial advancements in managing autistic children, the situation seems less debilitating compared to what it used to be in the past years. Adding neuroimaging advancements in the management can further improve the management of Autism Spectrum Disorder.

**Objectives:**

Our objective is to investigate structural changes in the insular cortex through our review of the available literature in the area of interest.

**Methods:**

Detailed literature search conducted using Pubmed, OVID, Google scholar with the search terms [insula] OR [autism] OR [brain changes] OR [ autism spectrum disorder] OR [insular cortex] OR [ insular changes] OR [neuroimaging] OR [neurology] OR [right insula] OR [left insula] OR [precentral cortex] OR[ amygdala] Or [emotion] Or[ memory] that produced around 300 results which were later narrowed down to be centered around search terms [autism] OR [insula] OR [structural changes] OR [brain]. 20 articles were made part of this review.

**Results:**

Results revealed that there are significant changes that are seen in neuroimaging of patients with Autism Spectrum Disorder. Their anterior cortex undergoes changes more than the posterior cortex with changes being more pronounced on the right side. Neuroimaging can be used to follow up with the prognosis of a chronic condition. Insula is a multifunctional region of the brain that is responsible for connecting cognitive, emotional, and movement functions in the brain. It is a highly functional area responsible for important neural connections. Insula is a highly emotion-sensitive area responsible for pain perception and emotion regulation. Insular changes can also help to diagnose the chronicity of the condition and age of patients with Autism.

**Conclusions:**

Cortical changes are visible on neuroimaging in several psychiatric conditions including schizophrenia, depression, anxiety, substance abuse, and alcoholism. Autism spectrum disorder is one of the diseases where neuroimaging can play an important role in planning further management. But unfortunately, this area is still under underresearched and needs to be given due importance to facilitate management of the chronic condition.

Keywords: cortex, insula, neuroimaging, autism spectrum disorder

**Disclosure of Interest:**

None Declared

